# Validation of two accelerated 4D flow MRI sequences at 3 T: a phantom study

**DOI:** 10.1186/s41747-019-0089-2

**Published:** 2019-02-26

**Authors:** Sebastian Ebel, Lisa Hübner, Benjamin Köhler, Siegfried Kropf, Bernhard Preim, Bernd Jung, Matthias Grothoff, Matthias Gutberlet

**Affiliations:** 1 Department of Diagnostic and Interventional Radiology, University of Leipzig – Heart Centre, Leipzig Strümpellstrasse 39, 04289 Leipzig, Germany; 2 Department of Simulations and Graphics, University of Magdeburg, Magdeburg, Germany; 3 Institute for Biometrics and Medical Informatics, University of Magdeburg, Magdeburg, Germany; 4 Department of Diagnostic, Interventional and Paediatric Radiology, University of Bern, Bern, Switzerland

**Keywords:** Four-dimensional (4D) flow, Magnetic resonance imaging, Reproducibility of results, Phantoms (imaging), Pulsatile flow

## Abstract

**Background:**

Four-dimensional (4D) flow magnetic resonance imaging (MRI) sequences with advanced parallel imaging have the potential to reduce scan time with equivalent image quality and accuracy compared with standard two-dimensional (2D) flow MRI. We compared 4D flow to standard 2D flow sequences using a constant and pulsatile flow phantom at 3 T.

**Methods:**

Two accelerated 4D flow sequences (GRAPPA2 and *k-t*-GRAPPA5) were evaluated regarding the concordance of flow volumes, flow velocities, and reproducibility as well as dependency on measuring plane and velocity encoding (*V*_enc_). The calculated flow volumes and peak velocities of the phantom were used as reference standard. Flow analysis was performed using the custom-made software “*Bloodline*”.

**Results:**

No significant differences in flow volume were found between the 2D, both 4D flow MRI sequences, and the pump reference (*p* = 0.994) or flow velocities (*p* = 0.998) in continuous and pulsatile flow. An excellent correlation (*R* = 0.99–1.0) with a reference standard and excellent reproducibility of measurements (*R* = 0.99) was achieved for all sequences. A *V*_enc_ overestimated by up to two times had no impact on flow measurements. However, misaligned measuring planes led to an increasing underestimation of flow volume and mean velocity in 2D flow accuracy, while both 4D flow measurements were not affected. Scan time was significantly shorter for *k-t*-GRAPPA5 (1:54 ± 0:01 min, mean ± standard deviation) compared to GRAPPA2 (3:56 ± 0:02 min) (*p* = 0.002).

**Conclusions:**

Both 4D flow sequences demonstrated equal agreement with 2D flow measurements, without impact of *V*_enc_ overestimation and plane misalignment. The highly accelerated *k-t*-GRAPPA5 sequence yielded results similar to those of GRAPPA2.

## Key points

- Both accelerated 4D flow sequences provided results not significantly different in comparison with the 2D flow sequence and the pump reference.
- Overestimation of velocity encoding did not impact on 4D flow accuracy
- Misaligned acquisition planes did not impact on 4D flow accuracy
- The highly accelerated *k-t*-GRAPPA5 sequence yielded results similar to those of GRAPPA2 in half the time.

## Background

Time-resolved three-dimensional (3D) phase-contrast magnetic resonance imaging (MRI) sequences, named four-dimensional (4D) flow sequences, represent an emerging technique for noninvasive evaluation of the cardiovascular system with full coverage of complete vessel systems such as the thoracic aorta [1, 2]. This technique gives new insights into physiological and pathophysiological flow patterns not currently observable with conventional two-dimensional (2D) flow sequences [3].

Similar to conventional 2D flow sequences, 4D flow sequences enables absolute quantification of flow parameters such as forward and backward flow volumes, flow velocities, and shunt volumes [4–6]. With 2D flow sequences, it is mandatory to perform measurements perpendicular to the longitudinal axis of any vessel of interest at the time of measurement. Misaligned measurements may lead to inaccurate results [7]. In contrast, in 4D flow isotropic data in all spatial directions can be obtained, making possible to create 3D reconstructions of every vessel within a given field of view. With these reconstructions, measurements should be independent of angulations. A further technical key setting in MRI flow measurements is a suitable choice of the velocity encoding (*V*_enc_). Overestimation of the velocities within the vessel can lead to inaccurate results, and underestimation of the *V*_enc_ can lead to phase wraps (aliasing) [6].

Most 4D flow data acquisitions with common navigator-gated sequences are time-consuming, requiring up to 25 min [8]. Therefore, recent developments aimed to shorten the acquisition time by using parallel imaging, advanced respiratory gating, and various strategies of undersampling [9–12].

At present, there are multiple vendors and research groups working on different strategies to reduce scan time and developing 4D flow sequences. With such a diversity of sequences, it seems to be difficult to create reproducible and valid datasets for clinical or research purposes and ultimately clinical applications, which underlines the importance of validation, evaluation, and standardisation of these novel sequences [8].

Thus, we planned a study using an MRI-compatible flow phantom in order to (1) compare two accelerated 4D flow sequences—a generalised autocalibrating partially parallel acquisition (GRAPPA) with acceleration factor 2 (GRAPPA2) and a recently introduced *k-t*-GRAPPA with acceleration factor 5 (*k-t*-GRAPPA5)—to a standard 2D flow sequence and the phantom setting for the accuracy of flow volume and velocity measurements and (2) elucidate the impact of different *V*_enc_ and misalignments of the measurement plane on 4D flow measurements.

## Methods

### MRI flow phantom

**Fig. 1:**
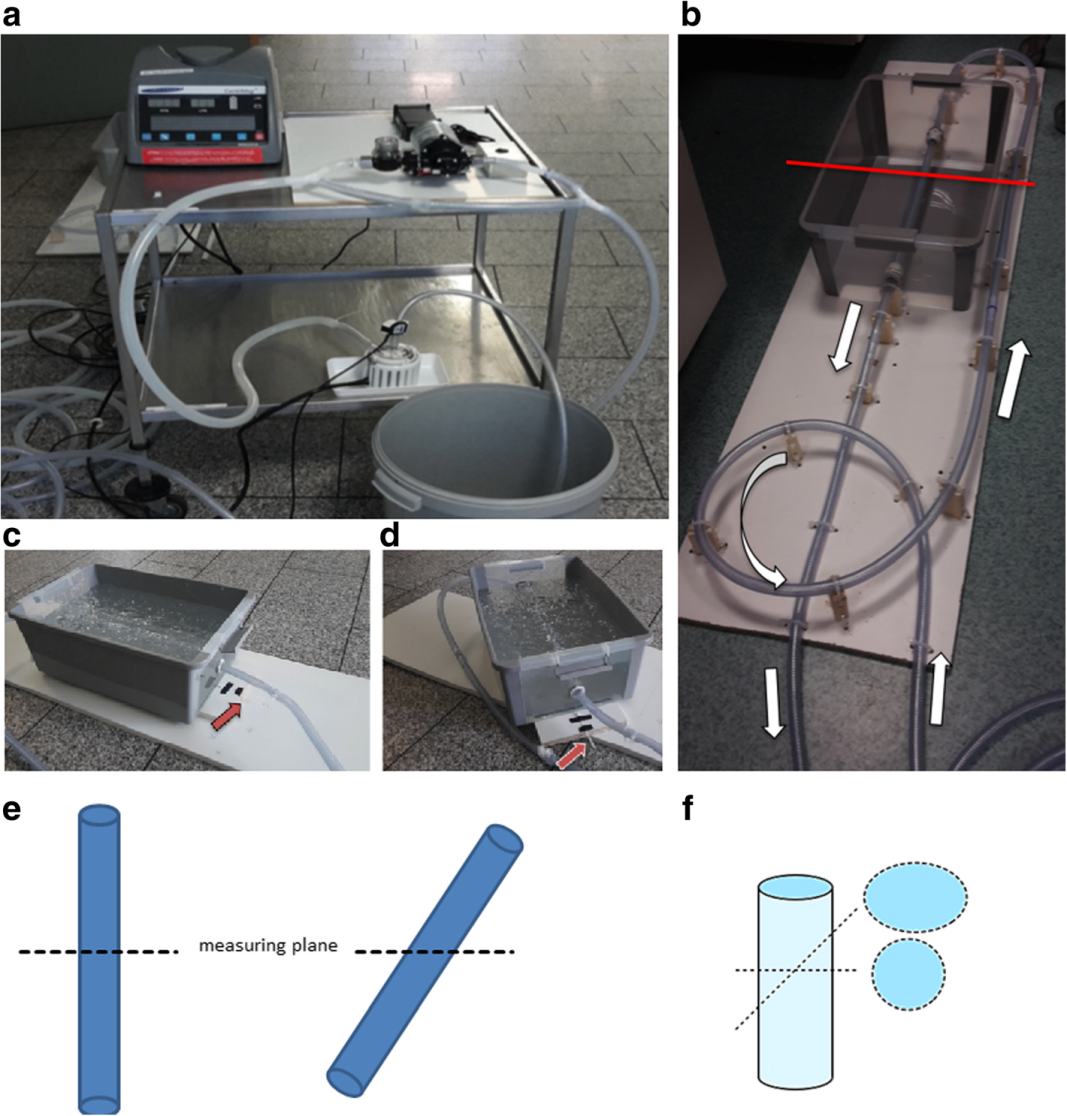
**a**, **b** Overview of the blood pump with control panel and inflow and outflow tubes. **c**, **d** The plastic tub filled with carbopol gel containing the tissue tube could be rotated. The red arrow indicates a goniometer. **c** Orthogonal orientation. **d** 45° rotation. **e** Schematic showing 2D flow measurements with the phantom vessel positioned along the *z*-direction of the MRI scanner (0°) and with an altered angle (45°). **f** Schematic changes of the cross-sectional area and shape

A custom-made flow phantom with a tube diameter of 0.5 in. was used. The straight fabric tube for the flow measurements was placed in a plastic tub filled with carbopol gel (Fig. 1), as previously described [13]. The fabric tube was arranged in the form of an open circuit, in which an additional plastic tub was embedded as a reservoir (see Fig. 1). The reservoir allowed bubble-free filling of the fabric tube with tap water and bubble-free operation of the open circuit flow phantom. A centrifugal blood pump CentriMag® (Thoratec, Pleasanton, CA, USA) was used to create constant flow. The pump allowed for setting rotational frequencies from 0 to 5,500 rotations per min (rpm). Measurements were performed with a rotational frequency between 1,500 and 5,500 rpm, with 500 rpm steps.

The blood pump was not MRI-conditional. Therefore, it had to be placed outside the magnetic field of the scanner. Since the centrifugal blood pump was gauged to blood and used in this experimental setting with tap water only, it was not possible to simply rely on the flow rates given by the user manual on specific rpms. Therefore, we had to perform volumetric measurements of the amount of water pumped through the circuit over a time period of 1 min for each used rpm. This given flow volume was used as the reference standard and to calculate the mean flow velocities within the tube (termed “pump reference”) with the following formula:

$$ v=Q/A $$

where *v* = flow velocity [cm/s], *Q* = flow volume [L/min]; *A* = cross-sectional area [cm^2^]

A roller pump of a heart-lung machine (Stöckert S3, Sorin Group, Munich, Germany) was used for pulsatile flow measurements. In the pulsatile flow experiments, 2D flow MRI (the current standard technique) was used as the reference standard. The plastic tub filled with carbopol containing the fabric tube was placed inside the MRI scanner in the isocentre of the magnetic field.

**Fig. 2:**
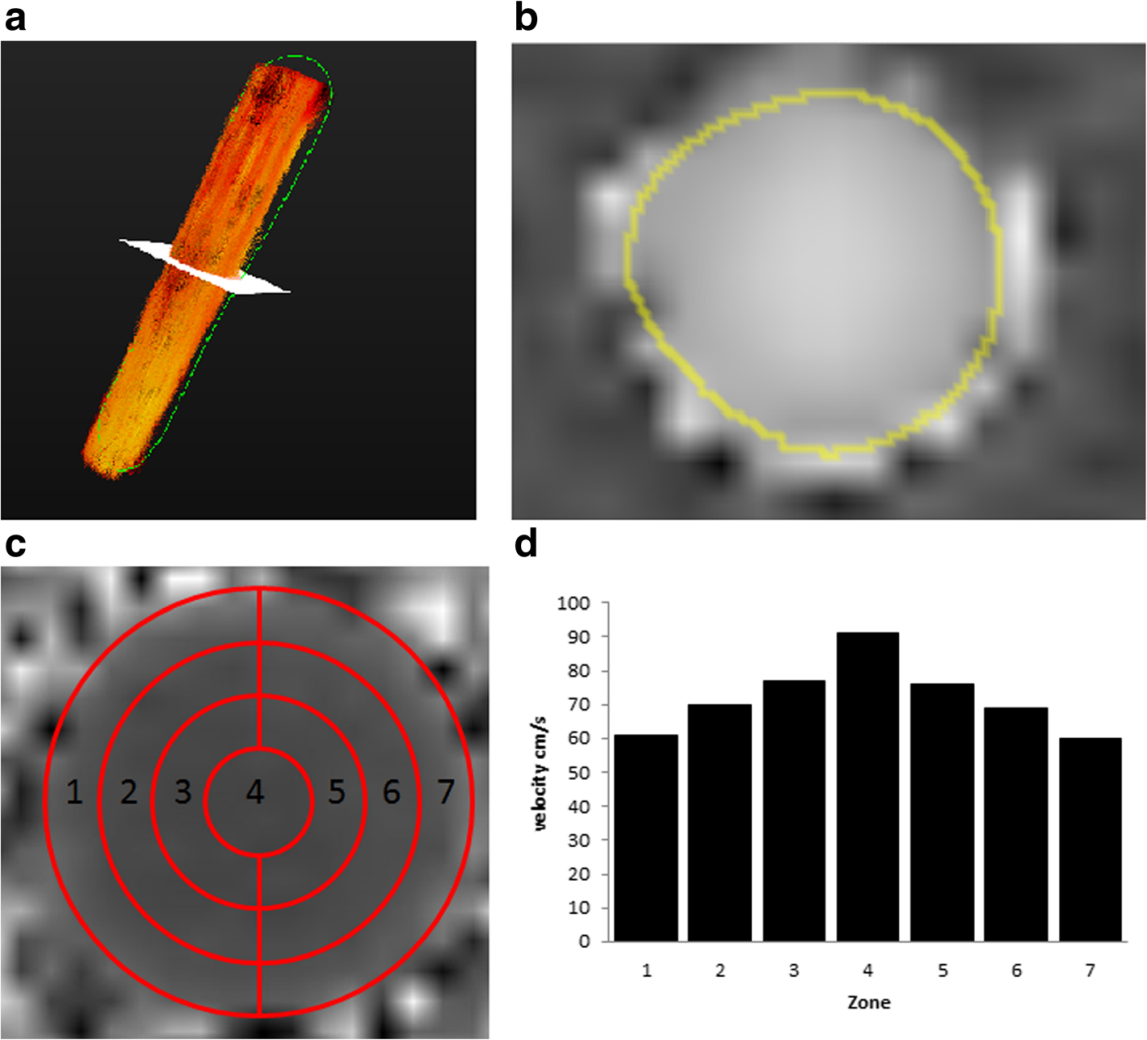
**a** Visualisation of constant flow inside the phantom vessel with time-resolved 3D pathlines (red). The yellow plane represents the measuring plane depicted in **b**. **b** Phase-contrast images obtained with the *k-t-*GRAPPA5 sequence showing a cross-section through the phantom vessel on the same level as in **a**. **c** Cross-section of the phantom vessel on a *k-t-*GRAPPA5 image subdivided into seven circular sectors. **d** Distribution of the flow velocities in the constant flow phantom within the seven circular sectors defined in **c** demonstrates a parabolic shape with higher flow velocities in the centre. cm/s, centimetres per second

The carbopol-filled plastic tub could be rotated and therefore allowed misalignment measurements between 0 and 45° in 15° steps (see Figs. 1 and 2).

### MR image acquisition

All studies were performed using a 3 T whole-body MRI system (Magnetom Verio Dot, Siemens Healthcare GmbH, Erlangen, Germany). A 16-channel anterior surface coil in combination with a 12-channel spine coil (Siemens Healthcare GmbH, Erlangen, Germany) was used.

All flow data were obtained with different *V*_enc_ (140, 160, 190, 220, 250, 280, and 350 cm/s in all directions, *i.e.* 4D) and the through-plane direction (2D). The first standardised 2D flow MRI acquisitions were performed in the middle of the fabric tube within the carbopol gel. The other sequence parameters were as follows: typical imaging parameters were spatial resolution 2.5 × 2.5 × 2.5mm^3^, TR/TE = 2.3 ms/1.8 ms, flip angle = 15°.

The 4D flow data were acquired in a 3D volume covering the whole fabric tube within the carbopol-filled plastic tub. First, data were acquired using standard parallel imaging (GRAPPA) with undersampling along the phase encoding (*k*_*y*_) direction with an acceleration factor 2 (GRAPPA2) [14]. Variable imaging parameters, such as the field of view and encoded phases, were kept constant for both 4D flow sequences. Next, *k-t*-accelerated 4D flow data (undersampling along *k*_*y*_, *k*_*z*_, and *t* dimensions) with an acceleration factor 5 (*k-t-*GRAPPA5) were obtained as reported by Jung et al. [2, 15].

Flow measurements need a time trigger for quantification purposes. In animals or humans, this triggering is achieved by electrocardiography or pulse triggering. The pulse wave could be used for triggering in our phantom study evaluating the pulsatile flow. Since such a trigger did not exist, in our constant flow phantom measurement, we had to use an electrocardiography simulator [16] (EKG Phantom 320, Müller & Sebastiani Elektronik GmbH, Munich, Germany), set to a constant high heart rate of 120 beats/min to accelerate the acquisition time.

The first series of measurements was performed with the flow phantom positioned along the *z*-direction of the scanner (0°). Afterwards, we changed the angle to 15°, 30°, and 45°. For the series of measurements with the angle greater than 0°, two 2D flow acquisitions had to be performed. The first acquisition was performed exactly perpendicular to the longitudinal axis of the fabric tube, *i.e.* an optimal “through-plane” flow measurement, with the second one being in an axial plane with its intersection through the phantom centre of rotation. 4D flow acquisition parameters were kept constant. All acquisitions were carried out twice (see Fig. 1 for details).

### Data analysis

#### Vessel segmentation, flow visualisation, and preprocessing

All processing and measurement steps were carried out using the custom-made software tool *Bloodline* [17–19]. The 3D reconstruction of the phantom was derived from temporal maximum intensity projections (TMIP). A centreline was drawn through the whole phantom semi-automatically beginning at the proximal end of the fabric tube. Flow within the phantom was visualised using temporal-resolved pathlines (Fig. 2a). We corrected for phase wraps, eddy currents, and background noise as reported previously. Eddy current corrections were performed using a technique with background subtraction [19, 20].

#### Measurements and flow quantifications

Measurements were carried out by two readers (> 4 years of experience in clinical 2D flow measurements). For quantification of the net flow (L/min) and peak velocity (cm/s), all 4D measuring planes were orientated perpendicular to the centreline of the phantom.

### Statistical analysis

All analyses were performed using MedCalc Statistical Software V15.11.4 (MedCalc Software, Ostend, Belgium). To compare the two 4D flow sequences with the reference standard, a two-way analysis of variance (ANOVA) with the sequences as the first factor and the respective varying model parameters as the second factor was used. If significant differences were found between groups for net flow, peak velocity, or image quality, the Dunnett test was performed to address the pairwise differences of the other measurements to the reference standard. A *p* value lower than 0.05 was considered to be significant. Correlation analyses were performed using scatter and linear regression analyses, as well as Bland-Altman plots. Bland-Altman analyses providing the mean differences between the measurements (bias), the standard deviation of the mean (SD), and the limits of agreement (LOA) were used for the different approaches to the flow analysis.

## Results

### Flow visualisation

For both constant and pulsatile flow, a parabolic flow profile within the phantom vessel with slower flow velocities in the peripheral layers and faster velocities in the centre of the vessel was demonstrated (Fig. 2c, d). However, the peak velocities were higher in pulsatile flow, as expected.

### Acquisition time

Mean acquisition time was 3:56 ± 0:02 min (mean ± SD) for the GRAPPA2 sequence and 1:54 ± 0:01 min for the *k-t-*GRAPPA5 (*p* = 0.002).

### Flow volumes quantification with constant flow

The first set of measurements was carried out with the centrifugal blood pump set to a constant rotational frequency of 1,500 rpm resulting in a flow volume of 1.7 L/min (pump reference).

**Fig. 3:**
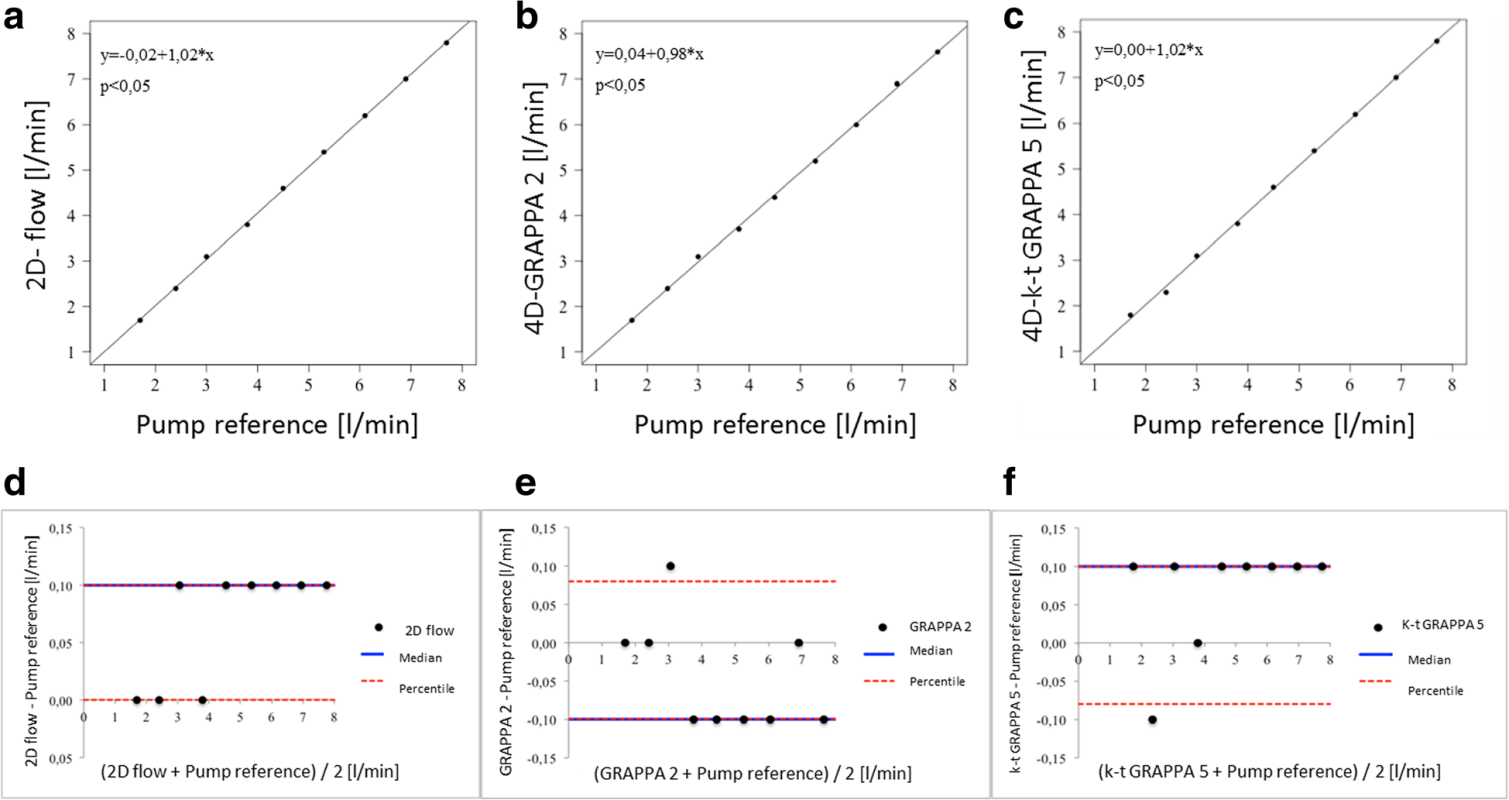
Scatter plots of the correlation between flow volumes [L/min] measured with (**a**) the 2D flow sequence, (**b**) the 4D flow GRAPPA2 sequence, and (**c**) the *k-t-*GRAPPA5 sequence versus the pump reference. The correlation coefficient *R* was 0.99 for each of the graphs. Bland-Altman analysis of flow volumes [L/min] measured with (**d**) the 2D flow sequence, (**e**) the 4D flow GRAPPA2 sequence, and (**f**) the *k-t-*GRAPPA5 sequence versus the pump reference the limits of agreement (LOA) between the flow volumes [L/min] in (**d**) 2D PCMRI, (**e**) GRAPPA2, and (**f**) *k*-*t*-GRAPPA5 and the pump reference. The limits of agreement were from 0.0 to 0.1, from − 0.1 to 0.08, and from − 0.08 to 0.1 L/min, respectively. 2D, two-dimensional; 4D, four-dimensional; GRAPPA, generalised autocalibrating partially parallel acquisition; L/min, litres per minute

Measurements in 2D flow and 4D flow showed very good agreements with mean flow volumes resulting to 1.7 ± 0.0 (mean ± SD) L/min for 2D flow sequences and 1.75 ± 0.1 L/min for both 4D flow GRAPPA2 and 4D *k-t-*GRAPPA5 sequences. The calculated mean flow volumes of 4D flow measurements slightly overestimated the volume flow compared to the standard reference and 2D flow measurements. However, there were no significant differences among the three measurements (*p* = 0.994), even though a large range of flow volumes was included, which was increased in 500 rpm steps from 1.7 to 7.7 L/min (Table 1). All 2D and 4D flow measurements demonstrated an excellent correlation with the standard reference with a correlation coefficient of *R* = 0.99 for all sequences. LOA were from 0.0 to 0.10 L/min for 2D flow, from -0.10 to 0.08 L/min for GRAPPA2, and from -0.08 to 0.1 L/min for *k-t-*GRAPPA5. Scatter plots and Bland-Altman analyses are shown in Fig. 3.

**Table 1:** Distribution of two measurements of the flow volume for 2D flow and two 4D flow phase-contrast sequences (GRAPPA2 and *k-t-*GRAPPA5) with different rotational frequencies of the blood pump

Rotational pump output, rpm	*V*_enc_, cm/s	Pump reference flow volume			2D flow PC flow volume			4D flow PC GRAPPA2 flow volume			4D flow PC *k-t-*GRAPPA5 flow volume		
		L/min		L/min, mean ± SD	L/min (difference from the pump reference)		L/min, mean ± SD	L/min (difference from the pump reference)		L/min, mean ± SD	L/min (difference from the pump reference)		L/min, mean ± SD
		Exam 1	Exam 2		Exam 1	Exam 2		Exam 1	Exam 2		Exam 1	Exam 2	
1,500	40	1.7	1.7	1.70 ± 0.0	1.7 (+ 0.0)	1.7 (+ 0.0)	1.70 ± 0.0	1.7 (+ 0.0)	1.8 (+ 0.1)	1.75 ± 0.1	1.8 (+ 0.1)	1.7 (+ 0.0)	1.75 ± 0.1
2,000	50	2.4	2.3	2.35 ± 0.1	2.4 (+ 0.0)	2.4 (+ 0.1)	2.40 ± 0.0	2.4 (+ 0.0)	2.4 (+ 0.1)	2.40 ± 0.0	2.3 (-0.1)	2.4 (+ 0.1)	2.35 ± 0.0
2,500	60	3.0	3.0	3.00 ± 0.0	3.1 (+ 0.1)	3.1 (+ 0.1)	3.10 ± 0.0	3.1 (+ 0.1)	3.1 (+ 0.1)	3.10 ± 0.0	3.1 (+ 0.1)	3.2 (+ 0.2)	3.15 ± 0.1
3,000	70	3.8	3.8	3.80 ± 0.0	3.8 (+ 0.0)	3.8 (+ 0.0)	3.80 ± 0.0	3.7 (-0.1)	3.8 (+ 0.0)	3.75 ± 0.1	3.8 (+ 0.0)	3.9 (+ 0.1)	3.85 ± 0.1
3,500	85	4.5	4.5	4.50 ± 0.0	4.6 (+ 0.1)	4.6 (+ 0.1)	4.60 ± 0.0	4.4 (-0.1)	4.5 (+ 0.0)	4.45 ± 0.1	4.6 (+ 0.1)	4.7 (+ 0.2)	4.65 ± 0.1
4,000	100	5.3	5.3	5.30 ± 0.0	5.4 (+ 0.1)	5.4 (+ 0.1)	5.40 ± 0.0	5.2 (-0.1)	5.3 (+ 0.0)	5.25 ± 0.1	5.4 (+ 0.1)	5.5 (+ 0.2)	5.45 ± 0.1
4,500	110	6.1	6.1	6.10 ± 0.0	6.2 (+ 0.1)	6.2 (+ 0.1)	6.20 ± 0.0	6.0 (-0.1)	6.2 (+ 0.1)	6.10 ± 0.1	6.2 (+ 0.1)	6.3 (+ 0.2)	6.25 ± 0.1
5,000	125	6.9	6.9	6.90 ± 0.0	7.0 (+ 0.1)	7.1 (+ 0.2)	7.05 ± 0.1	6.9 (+ 0.0)	6.9 (+ 0.0)	6.90 ± 0.1	7.0 (+ 0.1)	7.1 (+ 0.2)	7.05 ± 0.1
5,500	140	7.7	7.7	7.70 ± 0.0	7.8 (+ 0.1)	7.9 (+ 0.2)	7.85 ± 0.1	7.6 (-0.1)	7.7 (+ 0.0)	7.65 ± 0.1	7.8 (+ 0.1)	7.9 (+ 0.2)	7.85 ± 0.1

No significant differences between measurements and pump reference were observed (*p* = 0.994)

*2D* two-dimensional, *4D* four-dimensional, *cm/s* centimetres per second, *GRAPPA* generalised autocalibrating partially parallel acquisition, *L/min* litres per minute, *PC* phase-contrast, *SD* standard deviation of the mean, *V*_enc_ velocity encoding

### Flow velocities quantification with constant flow

#### Mean flow velocity

The centrifugal blood pump set to a rotational frequency of 1,500 rpm resulted in a mean velocity of 22 cm/s (pump reference). Measurements in 2D flow and 4D flow demonstrated very good agreements, with mean flow velocities of 22.5 ± 0.7 (mean ± SD) cm/s for the 2D flow sequence, 23 ± 0.0 cm/s for the 4D flow GRAPPA2 sequence, and 22.5 ± 0.7 cm/s for the 4D flow *k-t-*GRAPPA5 sequence. Mean flow velocities of 2D flow and 4D flow measurements demonstrated slightly higher values compared to the pump reference. However, no significant differences between the three measurements were observed (*p* = 0.998) (Table 2).

**Table 2:** Distribution of two measurements of the mean velocity for 2D flow and two 4D flow phase-contrast sequences (GRAPPA2 and *k-t-*GRAPPA5) with different rotational frequencies of the blood pump

Rotational pump output, rpm	*V*_enc_, cm/s	Pump reference mean velocity			2D flow PC mean velocity			4D flow PC GRAPPA2 mean velocity			4D flow PC *k-t-*GRAPPA5 mean velocity		
		cm/s			cm/s (difference from the pump reference)		cm/s	cm/s (difference from the pump reference)		cm/s	cm/s (difference from the pump reference)		cm/s
		Exam 1	Exam 2	Mean ± SD	Exam 1	Exam 2	Mean ± SD	Exam 1	Exam 2	Mean ± SD	Exam 1	Exam 2	Mean ± SD
1,500	40	22	22	22 ± 0.0	22 (+ 0)	23 (+ 0)	22.5 ± 0.7	23 (+ 1)	23 (+ 1)	23 ± 0.0	23 (+ 1)	22 (+ 0)	22.5 (± 0.7)
2,000	50	31	30	31 ± 0.7	32 (+ 1)	32 (+ 2)	32 ± 0.0	32 (+ 1)	32 (+ 2)	32 ± 0.0	31 (+ 0)	31 (+ 1)	31 (± 0.0)
2,500	60	39	39	39 ± 0.0	41 (+ 2)	41 (+ 2)	41 ± 0.0	41 (+ 2)	41 (+ 2)	41 ± 0.0	41 (+ 2)	42 (+ 3)	41.5 (± 0.7)
3,000	70	50	50	50 ± 0.0	50 (+ 0)	50 (+ 0)	50 ± 0.0	49 (-1)	50 (+ 0)	49.5 ± 0.7	50 (+ 0)	52 (+ 2)	51 (± 1.4)
3,500	85	59	59	59 ± 0.0	60 (+ 1)	60 (+ 1)	60 ± 0.0	58 (-1)	59 (+ 0)	58.5 ± 0.7	60 (+ 1)	62 (+ 3)	61 (± 1.4)
4,000	100	70	70	70 ± 0.0	71 (+ 1)	70 (+ 0)	70.5 ± 0.7	68 (-2)	70 (+ 0)	69 ± 1.4	71 (+ 1)	72 (+ 2)	71.5 (± 0.7)
4,500	110	80	80	80 ± 0.0	81 (+ 1)	82 (+ 2)	81.5 ± 0.7	79 (-1)	80 (+ 0)	79.5 ± 0.7	81 (+ 1)	83 (+ 3)	82 (± 1.4)
5,000	125	91	91	91 ± 0.0	92 (+ 1)	93 (+ 2)	92.5 ± 0.7	91 (+ 0)	91 (+ 0)	91 ± 0.0	92 (+ 1)	94 (+ 3)	93 (± 1.4)
5,500	140	101	101	101 ± 0.0	103 (+ 2)	103 (+ 2)	103 ± 0.0	100 (-1)	101 (+ 0)	100.5 ± 0.7	103 (+ 2)	104 (+ 3)	103.5 (± 0.7)

No significant differences between measurements and pump reference were observed (*p* = 0.998)

*2D* two-dimensional, *4D* four-dimensional, *cm/s* centimetres per second, *GRAPPA* generalised autocalibrating partially parallel acquisition; *PC* phase-contrast, *SD* standard deviation of the mean, *V*_enc_ velocity encoding

All 2D flow and 4D flow velocity measurements demonstrated an excellent correlation with the pump reference with a correlation coefficient of *R* = 0.99 for all sequences. LOA were from 0.0 to 2.0 cm/s for the 2D flow sequence, from -1.8 to 1.8 cm/s for the 4D flow GRAPPA2 sequence, and from 0.0 to 2.0 cm/s for the 4D flow *k-t-*GRAPPA5 sequence.

#### Peak velocity

The calculated peak velocities were identical for peak and mean velocity for all rpms. Measurements at 1,500 rpm (22 cm/s) resulted in a mean peak flow velocities of 35.0 ± 1.4 (mean ± SD) cm/s for the 2D flow sequence, 32.5 ± 0.7 cm/s for the 4D flow GRAPPA2 sequence, and 31.0 ± 0.0 cm/s for the 4D flow *k-t-*GRAPPA5 sequence. The measured mean peak flow velocities for 2D flow and 4D flow measurements demonstrated considerably higher values compared to the reference. The differences between all 2D flow or 4D flow measurements to the reference standard increased with higher rpms. However, no significant differences among the three measurements were observed (*p* = 0.999).

Despite the higher values, all 2D flow and 4D flow peak velocity measurements with GRAPPA2 and *k-t-*GRAPPA5 sequences demonstrated an excellent correlation with the reference standard with a correlation coefficient of *R* = 0.99 for all sequences. LOA were from -7.6 to -33.6 cm/s for the 2D flow sequence, from - 6.5 to -27.7 cm/s for the 4D flow GRAPPA2 sequence, and from -6.6 to -34.3 cm/s for the 4D flow *k-t-*GRAPPA5 sequence.

### Reproducibility

We repeated all measurements 4 weeks after the first set of measurements to evaluate the reproducibility of our results and found no significant deviations (Tables 2, 3, and 4). The flow volume LOA between the first and the second set of measurements were from 0.00 to 0.1 L/min for the 2D flow sequence, from 0.00 to 0.17 L/min for the 4D flow GRAPPA2 sequence, and from -0.06 to 0.1 L/min for the 4D *k-t-*GRAPPA5 sequence. The flow velocity LOA between the first and the second set of measurements were from -0.8 to 1.0 cm/s for the 2D flow sequence, from 0.00 to 1.8 cm/s for the 4D flow GRAPPA2 sequence, and from -0.8 to -1.0 cm/s 4D flow *k-t-*GRAPPA5 sequence.

**Table 3:** Distribution of measurements of the flow volumes, mean velocity, and peak velocity for 2D flow and two 4D phase-contrast sequences (GRAPPA2 and *k-t-*GRAPPA5) with different encoded velocities (*V*_enc_)

*V*_enc_, cm/s	Pump reference			2D flow PC			4D flow PC GRAPPA2			4D flow PC *k-t-*GRAPPA5		
	Flow volume, L/min	Mean velocity, cm/s	Peak velocity, cm/s	Flow volume, L/min	Mean velocity, cm/s	Peak velocity, cm/s	Flow volume, L/min	Mean velocity, cm/s	Peak velocity, cm/s	Flow volume, L/min	Mean velocity, cm/s	Peak velocity, cm/s
140	7.7	101	101	7.8	103	135	7.6	100	126	7.8	103	133
160	7.7	101	101	7.8	103	135	7.6	100	126	7.8	103	131
190	7.7	101	101	7.8	102	138	7.5	99	125	7.9	103	132
220	7.7	101	101	7.8	102	140	7.6	99	129	7.9	104	133
250	7.7	101	101	7.8	103	143	7.6	100	128	8.0	105	136
280	7.7	101	101	7.9	103	126	7.7	102	127	8.0	106	137
350	7.7	101	101	7.9	102	138	7.8	103	131	7.8	104	138

No significant differences between measurements and pump reference (*p* = 0.395)

*2D* two-dimensional, *4D* four-dimensional, *cm/s* centimetres per second, *GRAPPA* generalised autocalibrating partially parallel acquisition, *L/min* litres per minute, *PC* phase-contrast, *V*_enc_ velocity encoding

**Table 4:** Distribution of measurements of the flow volumes, mean velocity, and peak velocity for two different 2D flow phase-contrast acquisitions (orthogonal “through-plane” to the phantom vessel and misaligned) and two 4D flow phase-contrast sequences (GRAPPA2 and *k-t-*GRAPPA5) with angles between the longitudinal axis of the phantom vessel and the *z*-direction in 15° steps from 0 to 45°

Angle	Pump reference			2D flow PC			4D flow PC GRAPPA2			4D flow PC *k-t-*GRAPPA5		
	Flow volume, L/min	Mean velocity, cm/s	Peak velocity, cm/s	Flow volume, L/min	Mean velocity, cm/s	Peak velocity, cm/s	Flow volume, L/min	Mean velocity, cm/s	Peak velocity, cm/s	Flow volume, L/min	Mean velocity, cm/s	Peak velocity, cm/s
0°	5.3	70	70	5.4	71	93	5.4	71	90	5.4	71	96
15°	5.3	70	70	5.4	64	89	5.3	70	90	5.3	72	93
30°	5.3	70	70	5.1	60	82	5.3	70	87	5.3	70	87
45°	5.3	70	70	4.8	51	82	5.2	68	90	5.3	71	89

No significant differences between 4D flow measurements, orthogonal (“through-plane”) 2D flow measurements, and the pump reference (*p* = 0.999). In contrast, the misaligned 2D flow measurements demonstrated increasingly higher differences with the degree of misalignment

*2D* two-dimensional, *4D* four-dimensional, *cm/s* centimetres per second, *GRAPPA* generalised autocalibrating partially parallel acquisition, *L/min* litres per minute, *PC* phase-contrast, *V*_enc_ velocity encoding

### Impact of the velocity encoding on measurements

We repeated all acquisitions with the blood pump set to a constant rotational frequency of 5,500 rpm (resulting in a mean velocity of 101 cm/s) with different *V*_enc_ values of 140, 160, 190, 220, 250, 280, and 350 cm/s to evaluate the impact of the *V*_enc_ on our measurements. Data were analysed regarding the flow volume and mean and peak velocity, and we found no significant differences between the measurements taken with the optimal setting of 140 cm/s and all other settings (Table 3).

### Evaluation of different angles between the vessel and measuring plane

**Fig. 4:**
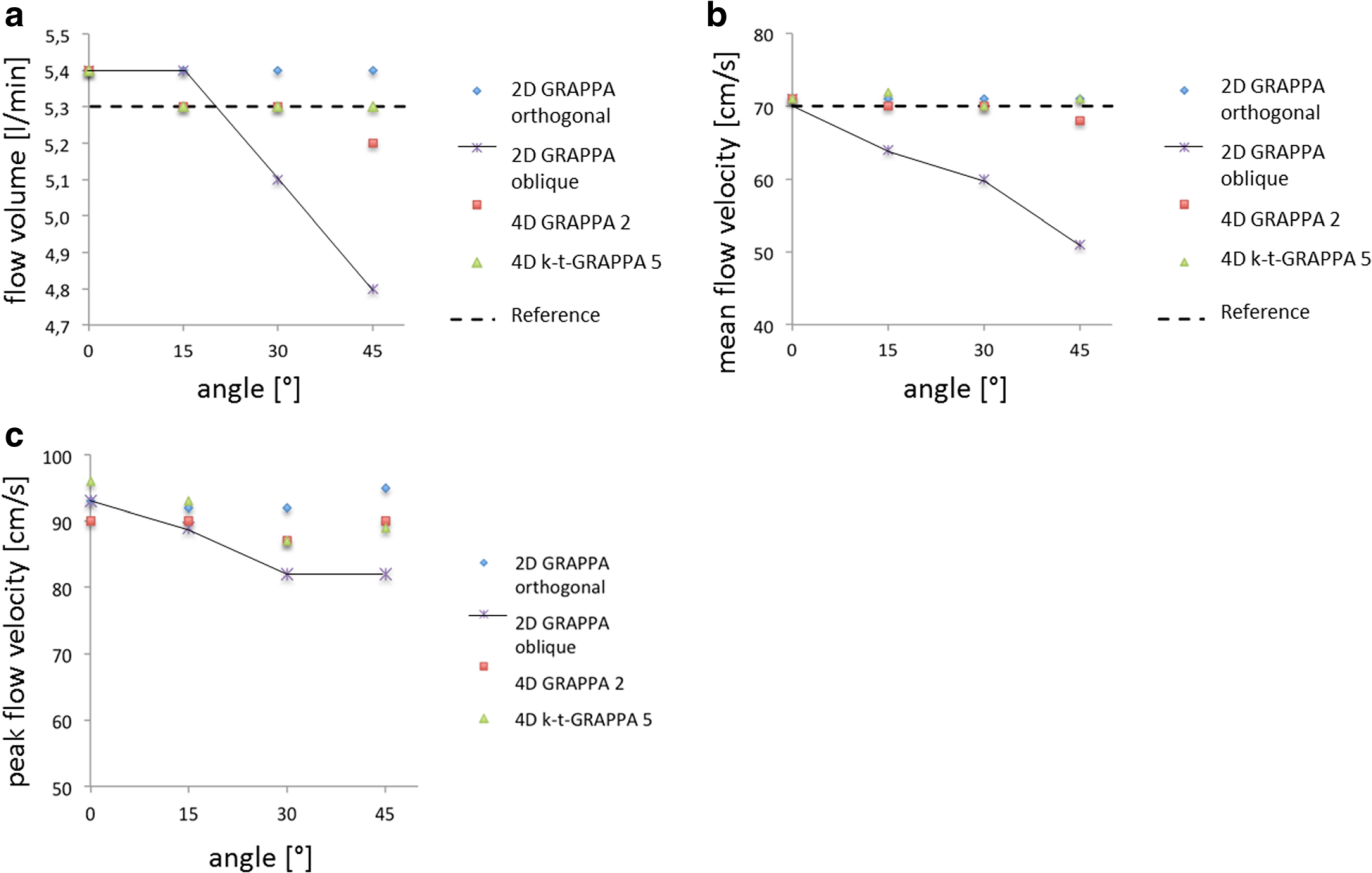
Influence of misalignment of the measuring plane on 2D flow and 4D flow measurements in constant flow regarding the (**a**) flow volume [L/min], (**b**) mean flow velocity [cm/s], and (**c**) peak flow velocity [cm/s]. 2D, two-dimensional; 4D, four-dimensional; cm/s, centimetres per second; GRAPPA, generalised autocalibrating partially parallel acquisition

To analyse the impact of different angles between the longitudinal axis of the phantom and the measuring plane, we used the following angles: 0°, 15°, 30°, and 45°. With increasing angles, the cross-sectional region of interest in 2D flow (the “through-plane” area) changed from a circular shape to an oval one (Fig. 1 e, f). As expected, we found an increasing underestimation of the flow volume and mean velocity in 2D flow with increasing deviation of the angle between the vessel and measuring plane, while we found no impact of angle changes on the measurements in both 4D flow sequences. We found significantly decreasing results regarding the peak velocity measured with 2D flow sequences (*p* = 0.006) and no impact on measurements with 4D flow sequences (*p* = 0.999 for both) (Table 4 and Fig. 4).

### Analysis of pulsatile flow

**Fig. 5:**
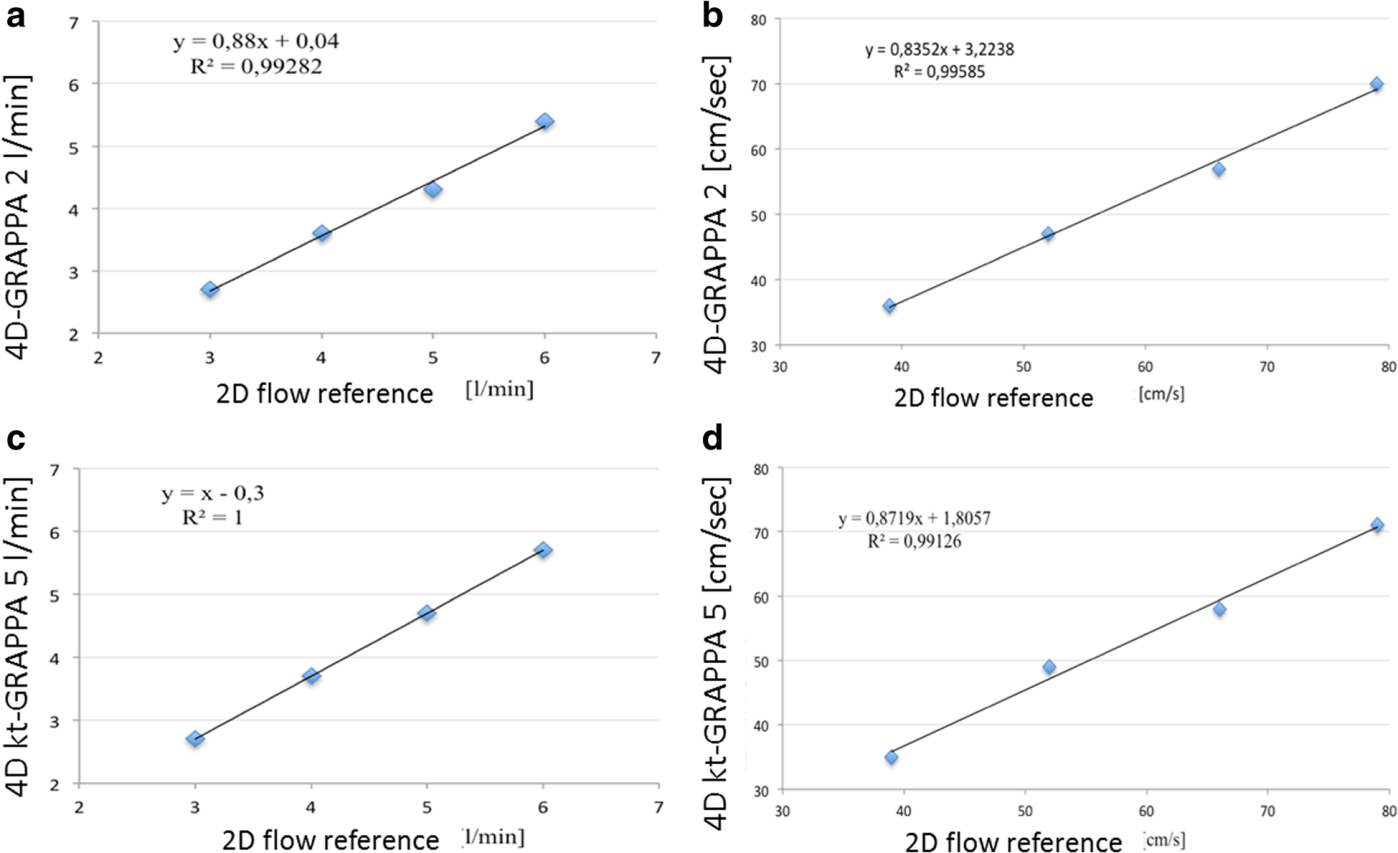
Scatter plots of the correlation between flow volumes and mean flow velocity measured with the 4D flow GRAPPA2 and *k-t-*GRAPPA5 sequences and the 2D flow sequence (reference) in pulsatile flow. **a**, **c** Flow volume [L/min]. **b**, **d** Mean flow velocity [cm/s]. 2D, two-dimensional; 4D, four-dimensional; cm/s, centimetres per second; GRAPPA, generalised autocalibrating partially parallel acquisition; L/min, litres per minute

We used 2D flow sequences as a reference standard for measurements with pulsatile flow, as explained in the “Methods” section. First, we found that in both constant and pulsatile flow, there was a parabolic-shaped flow profile (Fig. 2c, d). We performed multiple measurements with different settings of the blood pump resulting in a flow volume of from 3 to 6 L/min and a mean flow velocity from 40 to 80 cm/s. We found excellent correlations between 2D flow and 4D flow sequences (*R* = 0.99–1.0). The flow volume LOA between the 2D flow and the 4D flow GRAPPA2 sequences were from -0.18 to -0.38 and those between the 2D flow and the 4D flow *k-t-*GRAPPA5 were from -0.38 to 0.18 L/min. The mean velocity LOA between the 2D flow and the 4D flow GRAPPA2 sequences were from -1.63 to 2.13 cm/s and those between the 2D flow and the 4D flow *k-t-*GRAPPA5 were from -1.23 to 0.73 cm/s (Table 5 and Fig. 5).

**Table 5:** 4D flow measurements with GRAPPA2 and *k-t-*GRAPPA5 sequences of the flow volumes and mean velocities in pulsatile flow in comparison with 2D flow phase-contrast sequences

2D flow PC			4D flow PC GRAPPA2			4D flow PC *k-t-*GRAPPA5		
Flow volume, L/min	Mean velocity, cm/s	Peak velocity, cm/s	Flow volume, L/min	Mean velocity, cm/s	Peak velocity, cm/s	Flow volume, L/min	Mean velocity, cm/s	Peak velocity, cm/s
2.7	35	69	2.7	36	72	2.7	35	72
3.6	47	98	3.6	47	100	3.7	48	102
4.4	58	123	4.3	57	120	4.7	58	123
5.7	71	149	5.4	70	147	5.7	71	149
Correlation coefficient *R* (*p* value)*			1.0	1.0	1.0	0.99	0.99	0.99
Limits of agreement*			-0.18, 0.38	-1.63, 2.13	-5.8, 5.8	-0.38, 0.18	-1.23, 0.73	-5.8, 2.3

*2D* two-dimensional, *4D* four-dimensional, *cm/s* centimetres per second, *GRAPPA* generalised autocalibrating partially parallel acquisition, *L/min* litres per minute, *PC* phase-contrast

*Correlation coefficients and limits of agreement between the 4D flow PC GRAPPA2 and *k*-*t*-GRAPPA5 with the 2D flow PC sequences used as the reference standard

## Discussion

We achieved a significant scan time reduction by using the *k-t*-GRAPPA-accelerated 4D flow sequence compared to the GRAPPA2-accelerated 4D flow sequence without any impact on measurement results. We demonstrated a good correlation between the two different accelerated 4D flow sequences in a phantom study. This correlation applies for flow volumes, as well as for flow velocities, in both constant and pulsatile flow, and compared to phantom and 2D flow.

These results are consistent with the findings in the literature obtained using phase-contrast MRI in healthy volunteers and patients. In 2014, Schnell et al. [2] found a good correlation between a GRAPPA2-accelerated 4D flow sequence and a *k-t*-accelerated 4D flow sequence in healthy volunteers with different acceleration factors. In addition, these researchers found that a *k-t* acceleration factor 5 was most recommendable. Limitations of this study were the lack of validation against 2D flow sequences as the current standard of care and the lack of validation against a flow phantom.

We demonstrated a strong agreement (*R* = 0.99) between both accelerated 4D flow sequences and a standard 2D flow sequence. There are numerous articles regarding the validation of different 4D flow sequences against 2D flow sequences in different anatomical regions with good correlations [21–23] in humans. All of these studies lack a valid reference standard, such as invasive flow measurements or a phantom reference. In our study, we demonstrated not only a good correlation between both 4D flow sequences and 2D flow sequences but, more importantly, we also showed a good correlation between all flow sequences and the pump reference regarding the flow volume and mean velocity in constant and pulsatile flow. These basic evaluations of 4D flow sequences against a reliable reference, such as a flow phantom, are mandatory before including these new sequences in the clinical routine. Valid phantom studies also allow for performing consistency tests of the MRI systems to maintain and monitor acquired data quality, as is required for x-ray equipment.

We measured higher peak velocities compared to the ones calculated from the measured flow volumes of the pump reference in both accelerated 4D flow and in the 2D flow sequence. That approach was employed because even in constant flow, a parabolic flow profile exists [24] (see Fig. 2c, d) due to the surface tension of the water. This phenomenon means that the flow velocity in the peripheral layers is lower than in the centre of the vessel. The formula that we used for the calculation of peak flow velocities from the measured flow volume of the pump reference does not take such inhomogeneous flow profiles into account; therefore, the calculated results instead represent the mean flow velocity with the assumption of constant flow in all parts of the cross-sectional area of the tube than the peak flow velocity as it occurs in the centre of the vessel.

Several publications about the correlation between different 2D flow and 4D flow sequences with a flow phantom already exist, mostly using only constant flow [7, 23, 25, 26]. Nilsson et al. [26] found a good correlation between 2D flow, 4D flow, and the phantom reference with constant flow regarding the peak velocity and flow volumes. This finding fits with the results of our own study. However, the authors reported deviations between 2D flow and 4D flow regarding the velocity values from -2.3 to 13.0%. We found a slightly better correlation between 2D and 4D measurements (*R* = 0.99–1.0). In addition, we used a *k-t*-accelerated 4D flow sequence, which was not evaluated against a phantom.

However, flow phantoms with constant flow do not fully represent physiological flow patterns *in vivo*. In other words, a good correlation between measurements in 2D or 4D flow sequences and constant flow phantom measurements are a prerequisite but not synonymous with good correlations regarding the physiological pulsatile flow patterns. Therefore, a validation of the sequences against a flow phantom with pulsatile flow is mandatory. In our study, we showed excellent agreement (*R* = 0.99 and 1.0) between both accelerated 4D flow and 2D flow sequences regarding the different flow volumes and mean and peak velocities, and in physiological, pulsatile flow.

The number of phantom studies assessing 4D flow measurements of pulsatile flow remains limited. In our study, the correlations were slightly better than in the study of Garg et al. [27]. These researchers also compared different accelerated 4D flow sequences with a 2D flow sequence in a phantom study with pulsatile flow and found mean errors for 4D flow versus 2D flow from -3.2 to -8.8% for peak velocities. One possible reason for these differences might be that Garg and colleagues used a 1.5-T scanner, while we used a 3-T scanner. Imaging at higher field strengths provides a higher signal-to-noise ratio (SNR), which means increased image quality and accuracy of flow measurements [23, 28].

To obtain precise measurements with 2D flow sequences *in vivo*, it is important to place the measuring plane perpendicular to the vessel of interest. Oblique planes may lead to inaccurate measurements. Especially in congenital heart disease, proper positioning of the measuring planes can be difficult due to the altered anatomy. The results of this study underline one major advantage of 4D flow sequences: due to their 3D geometry, 4D flow sequences allow coverage of complete vessel systems, such as the thoracic aorta, with no need for special planning, and they allow for subsequent reconstruction and assessment of every vessel within the covered field of view. We showed that deviations of the acquisition planes had no impact on the accuracy of the measurements in 4D flow but led to inaccurate results in 2D flow measurements, depending on the degree of misalignment. In 2002, Lotz et al. [7] reported similar results using a flow phantom to evaluate 2D flow sequences. They found that oblique measuring planes in 2D flow sequences led to inaccurate measurements. To the best of our knowledge, this report was the first to demonstrate in a phantom flow study that deviations of the acquisition planes have no impact on the accuracy of 4D flow measurements. Therefore, the authors conclude that 4D flow is the ideal technique in regard to flow measurements in complicated vessel anatomy or in examinations of congenital heart disease with altered and complex anatomy.

One general rule for phase-contrast 2D and 4D flow measurements is that the better the *V*_enc_ fits the real velocity within the vessel of interest, the better and more accurate the measurements become [7]. While a *V*_enc_ set too low leads to phase wrapping, a *V*_enc_ set too high can lead to underestimation of the real flow velocities and volumes [7, 29, 30]. Underestimation occurs due to inadequate signal-to-noise ratio. Noise in the velocity encoding images increases with a higher *V*_enc_ [31]. In our study, we found no impact of a *V*_enc_ set too high on the accuracy of measurements in 2D flow as well as in 4D flow sequences. Even a *V*_enc_ set more than three times higher (350 cm/s, while the real velocity in the vessel was 101 cm/s) showed no effect. Again, one possible reason for these observations is that we performed all measurements using a 3-T scanner, while the previously mentioned studies by other groups were performed at 1.5 T only. Higher field strength means a higher SNR [28]. It is already known that phase-contrast imaging performed using 3-T scanners benefits from a better SNR relative to a 1.5-T scanner and could therefore be more suitable also for simultaneous examinations of the arterial and venous vessels.

Additionally, we also showed a good reproducibility of all measurements in all used 2D flow and 4D flow sequences, which is mandatory in regard to the integration of the sequences into the clinical routine, where patients may undergo numerous follow-up scans.

One limitation of this study is that we performed the evaluation only with a flow phantom with “healthy” vessels without any stenosis, as performed by other groups [27, 32, 33]. In addition, for the flow measurements, we did not use contrast medium to increase the SNR because our goal was to “simulate” *in vivo* conditions. In addition, we used a 3-T scanner, where the SNR is already high without the addition of contrast medium. Finally, although we found excellent correlations, a phantom cannot completely simulate physiological conditions. Therefore, these results are not completely transferable to *in vivo* settings. Proper evaluations in a phantom study, however, are an important prerequisite for evaluating these techniques in human volunteers or patients.

In conclusion, we showed that both 4D flow sequences and the 2D flow sequence used in this study provide accurate flow data when using a 3-T scanner. All sequences agreed strongly with the reference given by the flow phantom regarding the flow volumes and mean velocities in continuous and pulsatile flow. Importantly, we demonstrated that 4D flow sequences deliver accurate measurements even with misaligned acquisition planes, while there is a strong bias using the 2D flow sequences, enabling “fast-forward” planning. In addition, we showed that in a 3-T scanner, due to the high SNR, a *V*_enc_ set too high within a physiological range has no impact on the accuracy of measurements using 2D flow and 4D flow sequences in a phantom setting.

## Abbreviations

2D: Two-dimensional
3D: Three-dimensional
4D: Four-dimensional
GRAPPA: Generalised autocalibrating partially parallel acquisition
LOA: Limits of agreement
MRI: Magnetic resonance imaging
rpm: Rotations per min
SD: Standard deviation of the mean
SNR: Signal-to-noise ratio
*V*_enc_: Velocity encoding
